# Comparative Evaluation of Methods of DNA Extraction from Cryopreserved Bovine Semen for Molecular Diagnostic Applications

**DOI:** 10.3390/mps9030075

**Published:** 2026-05-09

**Authors:** Carlos A. Ramos-Jonapá, Lily X. Zelaya-Molina, Luis Felipe Guzmán, Edgar I. González-Jiménez, David Urbán-Duarte, Horacio Álvarez-Gallardo, Francisco J. Padilla-Ramírez

**Affiliations:** 1 Department of MIPPE, Universidad de Guadalajara, CUCBA Campus, Sistema de Postgrado, Camino Ing. Ramón Padilla Sánchez, 2100 Predio Las Agujas, Zapopan 45200, Jalisco, Mexico; javier.longino@gmail.com; 2 Centro Nacional de Recursos Genéticos-INIFAP, Av. Biodiversidad 2498, Tepatitlan de Morelos 47600, Jalisco, Mexico; lilyzelayam@yahoo.com.mx (L.X.Z.-M.); guzman.luis@inifap.gob.mx (L.F.G.); urban.david@inifap.gob.mx (D.U.-D.); alvarez.horacio@inifap.gob.mx (H.Á.-G.); 3 Centro Experimental Tecomán-INIFAP, Carretera Colima-Manzanillo, km 35, Tecomán 28930, Colima, Mexico; gonzalez.edgar@inifap.gob.mx

**Keywords:** bovine semen, semen cryopreservation, DNA extraction, molecular diagnostics, reproductive biotechnology

## Abstract

Cryopreserved bovine semen represents an accessible source of genetic material due to its widespread use in assisted reproductive technologies and the conservation of genetically valuable animals. However, DNA extraction from spermatozoa within this type of sample remains challenging due to the high protein content and the complex structure of the ejaculate, which can affect DNA yield and quality. The aim of this study was to identify and validate an efficient method for obtaining high-quality DNA from spermatozoa present in cryopreserved bovine semen for molecular diagnostic applications. Five DNA extraction protocols were evaluated: TRIzol™, MagMax™ Nucleic Acid Purification Kit, Rapid DNA™ Fecal/Soil Microbe Kit, a conventional phenol–chloroform protocol, and a modified phenol–chloroform–isoamyl alcohol protocol. All extracted genetic material was assessed by spectrophotometry (concentration and purity), and DNA integrity was evaluated by agarose gel electrophoresis. Statistical analysis revealed significant differences in DNA concentration among extraction methods (Friedman test, χ^2^ = 22.0, df = 4, *p* = 0.0002). Post hoc comparisons indicated that the modified phenol–chloroform–isoamyl alcohol protocol yielded significantly higher DNA concentrations compared to selected methods. This protocol showed the highest DNA concentration (1006.2 ± 829.4 ng/μL) and favorable purity values, and enabled consistent amplification in both conventional PCR and qPCR assays targeting the β-actin gene and *Tritrichomonas foetus*, respectively. These findings suggest that the modified protocol represents a suitable and promising approach for extracting genomic DNA from spermatozoa in cryopreserved bovine semen, with potential applications in molecular diagnostics and reproductive biotechnology.

## 1. Introduction

The use and commercialization of cryopreserved bovine semen play a central role in assisted reproductive technologies and genetic improvement programs within the livestock sector [1,2]. The availability of semen from genetically valuable animals has expanded its applications beyond reproduction, supporting molecular studies related to genetics, fertility, and population management [3]. Consequently, ensuring the quality of biological material derived from cryopreserved semen is essential for both productive and research purposes.

Molecular analyses based on DNA extracted from semen have gained increasing relevance in recent years. These analyses include pathogen detection, evaluation of sperm DNA integrity, identification of genes associated with fertility and semen quality, genetic improvement programs, and studies on genetic diversity, traceability, and conservation of bovine genetic resources [4,5,6,7]. The success of all these applications depends directly on the quality of the extracted genetic material, making DNA extraction a critical step in molecular workflows [8].

Multiple DNA extraction methods have been described, including organic extraction protocols, Chelex-based methods, salting out procedures, sucrose gradient separation, commercial kits, and magnetic bead-based systems [9,10]. Despite the availability of multiple DNA extraction protocols, their performance in cryopreserved bovine semen remains highly variable. Reported DNA yields and purity ratios differ considerably among studies, reflecting differences in sample composition, initial processing volumes, and extraction efficiency. For instance, phenol-based protocols have been associated with moderate purity values (A260/280 ranging from approximately 1.3 to 1.7), while commercial kits may yield inconsistent results depending on the presence of inhibitors. These discrepancies highlight the lack of consensus regarding the most suitable extraction approach for this biological matrix and underscore the need for standardized comparative evaluations. The efficiency of these methods is highly dependent on the biological matrix. Cryopreserved bovine semen represents a particularly complex substrate due to the high protein and sugar content of seminal plasma, the compact chromatin structure of spermatozoa, and the presence of cryoprotectants and diluents added during semen processing and storage in liquid nitrogen at −196 °C [11,12]. These characteristics can hinder cell lysis, reduce DNA yield, and introduce inhibitory compounds that compromise downstream molecular analyses [13].

Another factor influencing DNA recovery is the amount of initial biological material. In molecular assays, particularly for pathogen detection or low abundance targets, insufficient starting material or suboptimal extraction efficiency may result in unreliable amplification or false negative results [14]. Therefore, extraction protocols must ensure adequate DNA concentration, purity, and integrity to support conventional PCR and real-time PCR analyses [15].

The relevance of optimized DNA extraction methods is further emphasized by the increasing international movement of cryopreserved bovine semen and the need to comply with zoosanitary regulations, such as NOM-027-ZOO-1995 in Mexico, which establish sanitary requirements for semen from domestic animals and promote the implementation of laboratory-based diagnostic procedures to prevent the dissemination of reproductive pathogens [16]. Among these, *T. foetus* is of particular importance due to its impact on fertility and herd productivity, and its molecular detection commonly targets conserved regions such as the 5.8S rRNA gene [17,18].

Despite previous evaluations of DNA extraction protocols from bovine semen, direct comparisons among multiple methods performed under standardized experimental conditions, particularly using cryopreserved samples, remain scarce [19]. Therefore, the aim of this study was to compare different DNA extraction methods applied to cryopreserved bovine semen in order to identify a protocol capable of yielding genomic DNA with suitable concentration, purity, and integrity for downstream molecular applications.

## 2. Materials and Methods

### 2.1. Biological Samples

Cryopreserved bovine semen straws were obtained from the germplasm bank of the National Center for Genetic Resources (CNRG, Mexico; 20°52′24.3″ N, 102°42′38.6″ W). The study was conducted using six ejaculates obtained from six sexually mature bulls from the state of Jalisco, Mexico, (*n* = 6), which had been previously diluted with a soybean lecithin-based cryoprotectant to a final concentration of 20 × 10^6^ spermatozoa/mL, packaged in 0.25 mL plastic straws, and stored in liquid nitrogen at −196 °C.

For each DNA extraction protocol evaluated in this study, straws derived from the same six ejaculates, each originating from a different bull, were used to ensure biological comparability among methods. Each extraction method was applied to aliquots derived from these same ejaculates. However, due to methodological differences inherent to each protocol, initial processing volumes were not identical. Additionally, the sperm concentration was not re-quantified prior to DNA extraction, and therefore normalization based on spermatozoa number was not performed.

No semen collection or animal handling was performed as part of this study, as only archived cryopreserved samples were analyzed.

### 2.2. DNA Extraction

DNA extraction from cryopreserved bovine semen was performed using five different methods:

#### 2.2.1. TRIzol™ Reagent Method (Method A)

DNA extraction using TRIzol™ reagent (Invitrogen, Carlsbad, CA, USA) was performed with minor modifications to the manufacturer’s protocol for DNA isolation. Briefly, a 1.0 mL aliquot of cryopreserved bovine semen (4 straws of 0.25 mL) was thawed at room temperature (RT) and centrifuged at 12,000× *g* for 5 min. The resulting pellet was homogenized with 0.75 mL of TRIzol™ reagent and incubated at room temperature (RT) (20–25 °C) for 5 min to allow complete cell lysis. Subsequently, 0.2 mL of chloroform was added, and the mixture was incubated at RT for 3 min before centrifugation at 12,000× *g* for 15 min at 4 °C. Following phase separation, the aqueous phase was discarded, and DNA was recovered from the interphase and organic phase. To precipitate DNA, 0.3 mL of ethanol was added, mixed by gentle inversion, and incubated at RT for 3 min. The mixture was then centrifuged at 2000× *g* for 5 min at 4 °C, and the supernatant was discarded.

The DNA pellet was washed by resuspension in 1.0 mL of 0.1 M sodium citrate prepared in 10% ethanol and incubated at RT for 30 min with occasional gentle inversion, followed by centrifugation at 2000× *g* for 5 min at 4 °C. This washing step was repeated once. The pellet was then resuspended in 1.5 mL of 75% ethanol and incubated at RT for 20 min, followed by centrifugation at 2000× *g* for 5 min at 4 °C. The supernatant was carefully removed, and the pellet was air dried at RT for 10 min.

Finally, the DNA pellet was resuspended in 0.6 mL of 8 mM NaOH and homogenized by gentle pipetting. The suspension was centrifuged at 12,000× *g* for 10 min at 4 °C, the supernatant was discarded, and the final DNA pellet was resuspended in 50 μL of 1 mM EDTA (pH 8.0) and stored at −20 °C until further analysis.

#### 2.2.2. MagMax™ Core Nucleic Acid Purification Kit (Method B)

DNA extraction was performed using the MagMax™ Core Nucleic Acid Purification Kit (Thermo Fisher Scientific, Waltham, MA, USA) following the manufacturer’s instructions, with minor adaptations for cryopreserved bovine semen. Briefly, a 0.50 mL aliquot of cryopreserved semen (2 straws of 0.25 mL) was thawed at room temperature and centrifuged at 15,000× *g* for 2 min. From the resulting sample, 0.20 mL of supernatant was recovered for processing, following an adaptation of the manufacturer’s protocol for liquid biological samples.

The lysis step was carried out by adding 400 µL of Binding/Bead Mix (MagMax™ CORE Magnetic Beads combined with MagMax™ CORE Binding Solution) and 210 µL of PK/PBS Mix (MagMax™ CORE Proteinase K in 1× PBS). The mixture was incubated at 70 °C for 30 min and subsequently centrifuged (Serie BD AvantgardeLine, Germany). A volume of 600 µL of the lysate was recovered, combined with 420 µL of additional Binding/Bead Mix, and purified using a magnetic separation rack (DynaMag™ Magnet). DNA was finally eluted in 100 µL of the kit’s provided elution solution and stored at −20 °C until analysis.

#### 2.2.3. Quick-DNA™ Fecal/Soil Microbe Miniprep Kit (Method C)

DNA extraction was performed using the Quick-DNA™ Fecal/Soil Microbe Miniprep Kit (Zymo Research, Orange, CA, USA) according to the manufacturer’s instructions. A 1.0 mL aliquot of cryopreserved semen (4 straws of 0.25 mL) was thawed at RT and centrifuged at 15,000× *g* for 5 min. The supernatant was discarded, and the pellet was resuspended in 0.75 mL of ZR BashingBead™ Buffer.

The suspension was transferred to a ZR BashingBead™ lysis tube and centrifuged at 8000× *g* for 1 min. Subsequently, 1.2 mL of Genomic Lysis Buffer was added, and 0.80 mL of the mixture was loaded onto a Zymo-Spin™ IIC column. Following the washing steps, the column was placed in a clean 1.5 mL microcentrifuge tube, and DNA was eluted by adding 100 µL of DNA Elution Buffer directly to the column matrix and centrifuging at 10,000× *g* for 30 s. Eluted DNA was stored at −20 °C.

#### 2.2.4. Wilson’s Phenol–Chloroform (Method D)

DNA extraction was performed following the phenol–chloroform protocol described by Wilson [19]. Briefly, a 0.50 mL aliquot of cryopreserved bovine semen (2 straws of 0.25 mL) was thawed at room temperature, mixed with 0.50 mL of TE buffer (50:20), and centrifuged at 12,000× *g* for 5 min. The supernatant was discarded, and the pellet was resuspended with 10 µL of proteinase K (20 mg/mL) and 40 µL of 10% sodium dodecyl sulfate (SDS). The mixture was incubated at 56 °C for 2 h in a water bath (Thermo Scientific Max Q7000).

Subsequently, 0.50 mL of TE buffer (10:1) and 20 µL of 5 M NaCl were added. After homogenization, 0.50 mL of equilibrated cold phenol was added, vortexed at maximum speed, and centrifuged at 12,000× *g* for 5 min at 4 °C. The aqueous phase was transferred to a new tube and further purified by adding 0.50 mL of chloroform–isoamyl alcohol (24:1), followed by centrifugation at 12,000× *g* for 2 min at 4 °C. This step was repeated once.

DNA was precipitated by adding 0.50 mL of cold isopropanol and incubated at −20 °C for 17 h. Samples were then centrifuged at 12,000× *g* for 10 min at 4 °C, and the supernatant was discarded. The pellet was washed with 1.0 mL of cold 70% ethanol, centrifuged at 12,000× *g* for 1 min, air-dried at 65 °C for 2 h, resuspended in 50 µL of 1× TE buffer, and stored at −20 °C.

#### 2.2.5. Modified Green and Sambrook’s Phenol–Chloroform–Isoamyl Alcohol (Method E)

DNA extraction was performed using a modified version of the phenol–chloroform–isoamyl alcohol protocol described by Green and Sambrook [20]. A 1.0 mL aliquot of cryopreserved semen (4 straws of 0.25 mL) was thawed at room temperature and centrifuged at 4000× *g* for 10 min. The supernatant was discarded, and the pellet was washed twice with phosphate-buffered saline (PBS; pH 7.4).

For cell lysis, the pellet was resuspended in 0.20 mL of PBS and 1.0 mL of lysis buffer (Fecal Microbe/FastDNA™ Soil Microbe buffer supplemented with 100 µL of proteinase K, 20 mg/mL). The suspension was incubated at 55 °C for 5 h in a shaking water bath at 60 rpm.

For protein removal, the viscous lysate (approximately 1.5 mL) was divided into two 2.0 mL microtubes, and 0.50 mL of phenol:chloroform:isoamyl alcohol (25:24:1) was added to each. The samples were briefly vortexed at maximum speed and centrifuged at 12,000× *g* for 10 min. The supernatants were recovered, combined into a single tube, mixed with an equal volume of chloroform, and centrifuged at 9000× *g* for 10 min.

The aqueous phase was transferred to a new tube, and DNA was precipitated by adding 0.60 mL of isopropanol and 0.10 mL of 3 M sodium acetate, followed by incubation at −20 °C for 17 h. Samples were centrifuged at 9000× *g* for 10 min at 4 °C, and the pellet was washed with 1.0 mL of 70% ethanol. After air-drying at room temperature for 15 min, DNA was resuspended in 50 µL of 1× TE buffer and stored at −20 °C.

### 2.3. Evaluation of DNA Concentration, Purity and Integrity

DNA concentration and purity were determined using a NanoDrop™ 2000 spectrophotometer (Thermo Scientific, USA), with measurements configured for double-stranded DNA quantification. Sample purity was assessed by measuring absorbance ratios at A260/280 and A260/230.

DNA integrity was evaluated by agarose gel electrophoresis. Briefly, DNA samples were loaded onto a 1% (*w*/*v*) agarose gel prepared in 1× TAE buffer and stained with SYBR™ Safe DNA Gel Stain (Invitrogen). Electrophoresis was performed at 90 V for 45 min, and DNA bands were visualized under UV illumination using a gel documentation system (UVP Transilluminator, Analytik Jena, USA).

### 2.4. Functional Validation of Extracted DNA

Functional validation was performed using DNA extracted with the method that yielded the highest concentration, purity, and integrity. The suitability of the extracted DNA for downstream molecular applications was evaluated through amplification of a bovine endogenous reference gene (β-actin) by conventional endpoint PCR and detection of *T. foetus* by quantitative real-time PCR (qPCR).

For end-point PCR, amplification of the bovine β-actin gene was performed using a Select cycler thermocycler (Select Bioproducts, New York, NY, USA) in a final volume of 25.0 µL containing 1× buffer, 0.16 mM of each dNTP, 2.0 mM MgCL_2_, 0.3 µM of each forward (5′ ACTCGTACGTGGGGGATGAG 3′) and reverse (5′ TCCATGTCGTCCCAGTTGGTG 3′) primer, 1.5 U Taq polymerase, and 2.0 µL of DNA extracted from bovine semen at a concentration of 0.2 ng/µL. The amplification profile was 94 °C for 5 min, 35 cycles of 94 °C for 1 min, 64 °C for 45 s, 72 °C for 30 s, and 72 °C for 10 min.

Detection of *T. foetus* was performed using simplex qPCR on a StepOnePlus Real-Time PCR System with 96-well plates (MicroAmp^®^ Fast Optical 96-well Reaction Plate with barcode, 0.1 mL), targeting the conserved 5.8S rRNA gene. Reactions were prepared in a final volume of 20 µL containing 1× TaqMan™ Fast Advanced Master Mix (Applied Biosystems, USA), 0.3 µM of each forward (5′ TTCAGATAACGAGCGAGATTATCG 3′) and reverse (5′ GCTACCCTCTTCCTCCTGC 3′) primer, 0.18 µM of the TaqMan™ probe (5′-HEX-ACTCGTTTCTGTTTACAGAGATAGAGTTCT-TAMRA-3′), and 2.0 µL of DNA extracted from semen straws previously confirmed as positive for *T. foetus* at a concentration of 0.2 ng/µL. Each reaction was performed in triplicate. A no-template control (NTC), in which nuclease-free water replaced the DNA template, and a negative control (NC) consisting of DNA from *T. foetus*-negative samples were included in the run to monitor contamination and assay specificity. The qPCR amplification conditions consisted of an initial denaturation at 94 °C for 5 min, followed by 45 cycles of 94 °C for 30 s, 64 °C for 30 s, and 72 °C for 30 s.

Samples were considered positive when amplification curves crossed the threshold and produced Ct values within the expected range (approximately 14.7–15.8 cycles), consistent with efficient target detection.

### 2.5. Statistical Analysis

Statistical analysis was performed using R software version 4.5.1. Due to the repeated-measures design (same ejaculates analyzed across all extraction methods) and the non-normal distribution of the data, DNA concentration and purity parameters were analyzed using the Friedman test. When significant differences were detected, pairwise comparison were performed using Dunn’s post hoc test with Bonferroni correction. A significance level of *p* < 0.05 was considered.

## 3. Results

**Figure 1:**
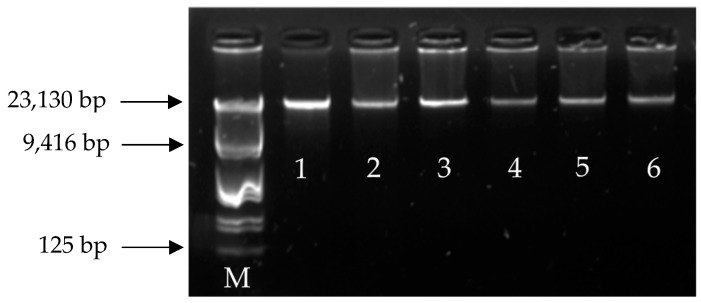
Agarose gel electrophoresis was used to evaluate the integrity of DNA extracted from cryopreserved bovine semen. Lambda DNA (λ) was used as a molecular weight marker (M). Lanes 1–6 correspond to DNA extracted using method E from six different samples, which exhibited the highest DNA concentration and purity.

DNA concentration and purity obtained using the five extraction methods are summarized in Table 1, while DNA integrity profiles of all analyzed samples are shown in Figure 1. Statistical analysis revealed significant differences in DNA concentration among extraction methods (Friedman test, χ^2^ = 22.0, df = 4, *p* = 0.0002).

Post hoc pairwise comparisons using Dunn’s test with Bonferroni correction showed that method E yielded significantly higher DNA concentrations compared to methods B (*p* = 0.00004) and C (*p* = 0.0299). Additionally, method A showed significantly higher DNA concentration than method B (*p* = 0.0087). No significant differences were observed between method E and methods A and D (*p* > 0.05), although a trend toward higher values was observed. The highest DNA concentrations were obtained with methods A and E, yielding mean values of 132.8 ± 116.0 ng/µL and 1006.2 ± 868.51 ng/µL, respectively. In contrast, the lowest DNA concentration was observed with method B (3.1 ± 1.4 ng/µL), which may be attributed to the lower efficiency of cell lysis and incomplete disruption of sperm chromatin, resulting in reduced DNA recovery from this method.

Regarding DNA purity, method E produced the most favorable absorbance ratios, with mean A260/280 and A260/230 values of 1.9 ± 0.02 and 2.0 ± 0.11, respectively. Method C showed elevated A260/280 values (4.50 ± 6.72), whereas method D exhibited low A260/230 ratios (0.02 ± 0.01), suggesting potential contamination or measurement artifacts. Variability among extraction methods was observed for both concentration and purity parameters.

The assessment of DNA integrity by agarose gel electrophoresis revealed that DNA extracted using method E displayed intact profiles with well-defined bands and higher signal intensity, indicating better preservation of genomic DNA integrity (Figure 1).

**Figure 2:**
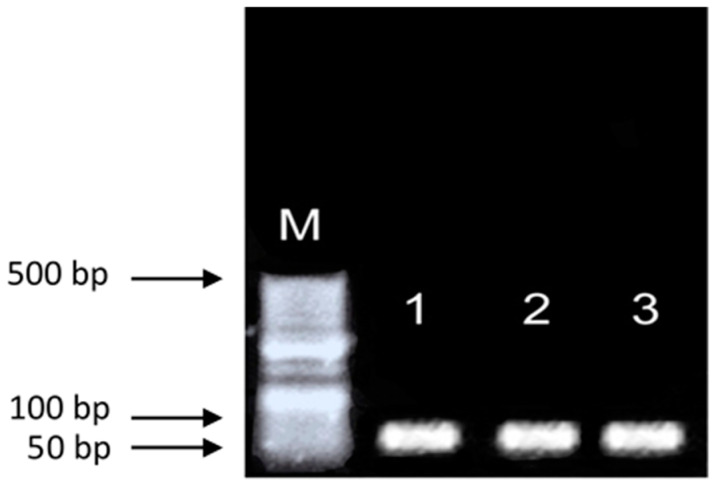
Amplification of the bovine β-actin gene by conventional PCR, showing the expected 74 bp fragment (lanes1-3). A 50 bp DNA molecular weight marker (M) digested with HindIII was used as a size reference.

**Figure 3:**
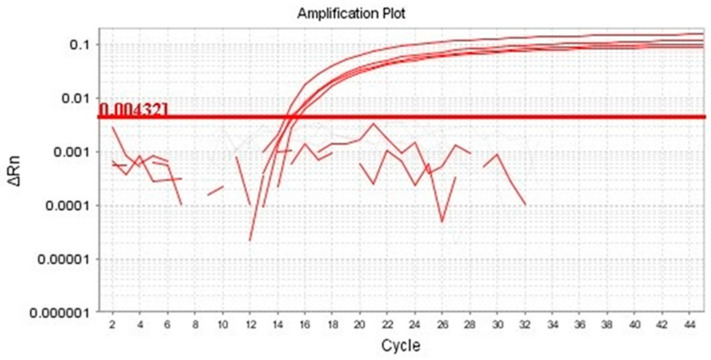
Amplification of the 5.8S rRNA gene of *T. foetus* by quantitative real-time PCR. Positive samples (red lines) showed clear amplification curves crossing the threshold within the expected Ct range (14.79–15.79).

Functional validation assays were conducted using DNA extracted with method E. Amplification of the bovine endogenous reference gene β-actin was successfully achieved by conventional PCR, yielding the expected 74 bp fragment (Figure 2). In addition, the presence of *T. foetus* was detected by quantitative real-time PCR through amplification of the 5.8S rRNA gene in semen straws previously confirmed as positive for the pathogen. qPCR analysis confirmed successful amplification of the target gene using DNA extracted with method E. Ct values shown in Table 2 ranged from 14.79 to 15.79, indicating efficient amplification and high target DNA abundance (Figure 3).

## 4. Discussion

DNA extraction from cryopreserved bovine semen remains a methodological challenge due to the physicochemical characteristics of this biological matrix. Semen is rich in proteins, sugars, and other compounds that can interfere with DNA purification and inhibit enzymatic reactions, particularly those based on PCR. In addition, the highly compacted chromatin structure of spermatozoa limits DNA release and contributes to variability in extraction efficiency. Previous studies have reported lower DNA concentrations than those obtained in the present work, although direct comparisons are often complicated by differences in extraction protocols and the initial volume of semen used [21,22]. In this study, DNA was extracted from initial volumes ranging from 0.5 to 1.0 mL of cryopreserved semen, which may partially explain the higher yields observed. However, successful DNA extraction from substantially smaller semen volumes, as low as 0.010 mL, has also been reported [23], indicating that DNA yield is influenced not only by sample volume but also by protocol design and lysis efficiency.

Among the evaluated methods, the modified Green and Sambrook phenol–chloroform–isoamyl alcohol protocol (method E) demonstrated superior performance in terms of DNA yield and purity. An A260/280 ratio of approximately 1.8–2.0 is generally considered indicative of pure DNA. The A260/280 and A260/230 ratios obtained in this study were consistent with or higher than those reported for standardized phenol-based and commercial extraction methods used in bovine semen. For example, previous studies have reported lower purity ratios, including A260/280 values around 1.32–1.75 and A260/230 values below 1.0 in semen-derived DNA extracts [21,24], whereas the values obtained with method E in the present work (approximately 1.9 and 2.0, respectively) indicate improved removal of protein and chemical contaminants.

Commercial extraction methods showed heterogeneous performance. Although the TRIzol™-based protocol (method A) yielded relatively high DNA concentrations, purity values were suboptimal, indicating the persistence of contaminants. Similarly, commercial extraction systems such as TRIzol™ have been widely applied in reproductive tissues; however, they frequently yield DNA with suboptimal purity ratios, particularly with respect to A260/230, which may compromise downstream enzymatic applications [25]. In contrast, the optimized protocol evaluated in this study provided both higher DNA concentrations and more consistent purity metrics, suggesting improved efficiency in sperm chromatin disruption and contaminant removal.

A limitation of this study is that DNA extraction protocols were performed using different initial processing volumes and without normalization based on spermatozoa number. Although the same ejaculates were used across all methods, these differences may have influenced DNA yield and should be considered when interpreting the comparative performance of the evaluated protocols.

Functional validation was restricted to the method that met minimum requirements for DNA concentration and purity, as these parameters are critical for downstream molecular applications. However, it cannot be excluded that other methods may yield amplifiable DNA under certain conditions, and further evaluation would be required to fully assess their functional performance.

DNA integrity analysis further highlighted clear differences among extraction methods. Pronounced degradation and smearing were observed in samples extracted using method B, whereas intact and well-defined DNA bands were consistently obtained with method E, corresponding to the phenol–chloroform-based protocol. The wide range of DNA concentration values observed for this method may be attributed to factors such as variability in semen processing, differences in spermatozoa concentration among ejaculates, and intrinsic sample quality, including the physiological condition or health status of the donor at the time of collection. Preservation of DNA integrity is particularly important for PCR- and qPCR-based assays, as fragmented DNA or co-extracted inhibitors can negatively affect amplification efficiency and reproducibility, potentially leading to false-negative results [26,27]. The combination of high yield, optimal purity ratios, and preserved integrity supports the suitability of the modified phenol–chloroform protocol under the conditions evaluated in this study for processing cryopreserved bovine semen, a biological matrix characterized by high protein content and the presence of PCR inhibitors [27].

The functional suitability of the extracted DNA was confirmed through successful amplification of the bovine β-actin gene by conventional PCR and detection of *T. foetus* by qPCR targeting the 5.8S rRNA gene. These results suggest that DNA obtained using method E is compatible with both endpoint and real-time PCR assays and was free of inhibitory substances. This is particularly relevant for pathogen detection in semen samples, where the amount of pathogen-derived genetic material is often low relative to host DNA, and extraction efficiency plays a critical role in assay sensitivity [28,29]. The use of the 5.8S rRNA gene as a molecular target for *T. foetus* detection is well supported, given its conserved nature and diagnostic reliability in trichomonas identification [29].

Cryopreserved bovine semen is recognized as a potential vehicle for the transmission of a wide range of bacterial and viral pathogens of reproductive importance, including agents associated with infertility, early embryonic loss, and restrictions on international trade [30,31]. In this context, the performance of the optimized extraction protocol supports its potential applicability for molecular diagnostics and zoosanitary surveillance programs, as required for the control of diseases associated with artificial insemination and semen trade. Reliable DNA extraction represents a fundamental step for compliance with sanitary regulations governing the movement and commercialization of bovine semen, as emphasized by international guidelines and national regulatory frameworks [32,33].

Beyond its diagnostic relevance, the extraction of high-quality DNA from cryopreserved semen has broader applications in reproductive biotechnology and animal genetics. DNA obtained using the optimized protocol may be suitable for studies on fertility-related genes, semen quality, genetic improvement programs, and analyses of genetic diversity, traceability, and conservation of bovine genetic resources [34,35]. These applications are particularly relevant for the study and preservation of local and creole cattle populations, where access to high-quality biological material is often limited and semen banks represent a valuable genetic reservoir [36]. These findings indicate that, under the conditions evaluated, the modified phenol–chloroform protocol not only outperforms other methods tested in this study but also compares favorably with previously reported standardized extraction approaches for bovine semen, supporting its applicability for molecular diagnostics and reproductive biotechnology applications.

## 5. Conclusions

Based on the comparative evaluation of DNA extraction methods applied to cryopreserved bovine semen, the modified Green and Sambrook phenol–chloroform–isoamyl alcohol protocol demonstrated the best overall performance in terms of DNA concentration, purity, and integrity among the evaluated methods. This protocol consistently produced high-quality DNA suitable for downstream molecular applications, including PCR- and qPCR-based assays, highlighting its efficiency for processing a challenging biological matrix such as cryopreserved semen.

In contrast, the TRIzol™ reagent method yielded lower purity values, although it provided DNA concentrations that may still be adequate for certain molecular applications. However, its performance was inferior in comparison with the optimized phenol–chloroform protocol.

Although functional validation was performed using the best-performing method, and differences in initial sample processing may have influenced DNA yield, the results collectively indicate that the modified phenol–chloroform method improves DNA recovery and quality under the conditions evaluated. Therefore, this protocol represents a reliable and efficient alternative for DNA extraction from bovine semen, with potential applications in molecular diagnostics and reproductive biotechnology.

## Figures and Tables

**Table 1:** DNA concentration and purity obtained from cryopreserved bovine semen using five different extraction methods.

DNA Quality Parameters	Sample	DNA Extraction Method				
		(A)	(B)	(C)	(D)	(E)
**Concentration (ng/μL)**	1	364.7	1.1	266.0	23.3	2488.9
	2	121.9	3.1	20.3	27.1	1643.4
	3	96.4	2.2	9.1	22.8	569.6
	4	91.7	3.0	33.1	17.1	561.8
	5	65.5	4.6	3.1	21.4	463.7
	6	56.4	5.0	19.4	27.2	310.0
	Mean ± SD	132.8 ± 106.3	3.1 ± 1.5	58.5 ± 98.6	23.1 ± 3.4	1006.2 ± 829.4
**A260/280 ratio**	1	1.7	1.3	1.8	1.8	1.9
	2	1.3	1.0	1.7	1.7	1.9
	3	0.3	1.1	1.6	1.8	1.8
	4	1.7	1.1	1.3	2.0	1.8
	5	1.3	1.6	18.2	1.7	1.8
	6	1.8	1.7	2.0	1.9	1.8
	Mean ± SD	1.3 ± 0.5	1.3 ± 0.2	4.5 ± 6.6	1.90 ± 0.09	1.9 ± 0.01
**A260/230 ratio**	1	0.8	0.6	0.9	0.03	2.1
	2	0.7	0.3	1.9	0.03	2.2
	3	0.6	0.3	0.2	0.03	2.0
	4	0.7	0.4	0.5	0.02	2.0
	5	0.6	0.3	0.03	0.02	2.0
	6	0.8	0.3	0.1	0.03	1.8
	Mean ± SD	0.7 ± 0.08	0.4 ± 0.1	0.6 ± 0.7	0.03 ± 0.005	2.0 ± 0.1

SD: standard deviation.

**Table 2:** Quantitative PCR (qPCR) Ct values, controls, and interpretation criteria for target detection.

Sample	Target	Ct	Interpretation
NTC	5.8S rRNA	NA	Negative
NC	5.8S rRNA	NA	Negative
PC	5.8S rRNA	14.79	Positive
1	5.8S rRNA	15.19	Positive
1	5.8S rRNA	15.22	Positive
1	5.8S rRNA	15.79	Positive

PC: positive control; NC: negative control; NTC: no template control.

## Data Availability

The data used to support the findings of this study are available upon request from the corresponding author.

## Abbreviations

The following abbreviations are used in this manuscript:

SDS	Sodium dodecyl sulfate
AI	Artificial Insemination
RT	Room Temperature
EDTA	Ethylenediaminetetraacetic acid
TE	Tris-EDTA buffer
